# A Novel Impedance Biosensor for Measurement of Trans-Epithelial Resistance in Cells Cultured on Nanofiber Scaffolds

**DOI:** 10.3390/bios7030035

**Published:** 2017-08-31

**Authors:** Robert A. Schramm, Matthew H. Koslow, Deirdre A. Nelson, Melinda Larsen, James Castracane

**Affiliations:** 1 Colleges of Nanoscale Science and Engineering, SUNY Polytechnic Institute, 257 Fuller Road, Albany, NY 12203, USA; 2 Department of Biological Sciences, University at Albany, State University of New York, 1400 Washington Avenue, Albany, NY 12222, USA; mkoslow@albany.edu (M.H.K.); dnelson@albany.edu (D.A.N.); mlarsen@albany.edu (M.L.)

**Keywords:** scaffold, impedance, non-destructive testing, trans-epithelial electrical resistance, nanofibers, barrier function, epithelial cells, salivary gland

## Abstract

Nanofibrous scaffolds provide high surface area for cell attachment, and resemble the structure of the collagen fibers which naturally occur in the basement membrane and extracellular matrix. A label free and non-destructive method of assessing the interaction of cell tissue and scaffolds aids in the ability to discern the effective quality and magnitude of any scaffold modifications. Impedance cell spectroscopy is a biosensing method that employs a functional approach to assessing the cell monolayer. The electrical impedance barrier function of a cell monolayer represents the level of restriction to diffusion of charged species between all adjacent cells across an entire contiguous cellular monolayer. The impedance signals from many individual paracellular pathways contribute to the bulk measurement of the whole monolayer barrier function. However, the scaffold substrate must be entirely porous in order to be used with electrochemical cell impedance spectroscopy (ECIS) and cells must be closely situated to the electrodes. For purposes of evaluating cell-scaffold constructs for tissue engineering, non-invasive evaluation of cell properties while seeded on scaffolds is critical. A Transwell-type assay makes a measurement across a semi-permeable membrane, using electrodes placed on opposing sides of the membrane immersed in fluid. It was found that by suspending a nanofiber scaffold across a Transwell aperture, it is possible to integrate a fully functional nanofiber tissue scaffold with the ECIS Transwell apparatus. Salivary epithelial cells were grown on the nanofiber scaffolds and tight junction formation was evaluated using ECIS measurements in parallel with immunostaining and confocal imaging. The trans-epithelial resistance increased coordinate with cell coverage, culminating with a cell monolayer, at which point the tight junction proteins assemble and strengthen, reaching the peak signal. These studies demonstrate that ECIS can be used to evaluate tight junction formation in cells grown on nanofiber scaffolds and on effects of scaffold conditions on cells, thus providing useful biological feedback to inform superior scaffold designs.

## 1. Introduction

This research investigated a continuous, non-destructive method of characterizing the function of the cell–cell junctions in continuous epithelial cell line monolayers. Electrochemical cell impedance spectroscopy (ECIS) is a technique that enables real-time electrical detection of the trans-epithelial electrical resistance (TEER) of a cell monolayer [1]. This technique requires the placement of electrodes above and below a Transwell membrane, and the passing of alternating current, of frequencies between 40 Hz and 100,000 Hz, across the suspended membrane, all the while immersed in media. A potentiostat applies a continuous sinusoidal voltage to the active electrode, while the opposite electrode remains grounded, which imparts a force upon charged, dissolved species within the cellular media causing them to diffuse towards the electrode of opposite charge. At low frequencies (40 Hz), the charged species have enough time before the next cycle to percolate slowly through the cell membrane at the point of minimum resistance. Traversing a cell membrane and entering the cytoplasm is energetically unfavorable and, therefore, charged species diffuse primarily between adjacent cells through the paracellular pathways [2]. The lock-in amplifier circuitry in the ECIS system allows for precise signal deconvolution of the resistance from the complex impedance, separating it from the capacitive element. This enables the active, direct probing of the permeability of the pathway (barrier function) without disturbing the living cells. Traditionally, these assays are performed by seeding cells on a thin, porous membrane in a Transwell assembly and allowing a monolayer to form, which retains a free permeable pathway through the membrane and monolayer to take electrical impedance measurements [3]. TEER measurement also reduces the likelihood of misinterpreting scaffold characteristics because of non-representative point-of-interest selection, which can occur during microscopy, by generating an average measurement over many cell junctions throughout the cell monolayer. The core benefit of using this technique is that the assay is non-destructive, and maintains active and undisturbed cell growth throughout the experiment. This opens up more types of investigation, and can explore even transient effects of scaffolds on cell behavior, other than molecular expression levels and localization, that are possible with destructive endpoint assays.

Damage to the salivary gland tissue in humans can be caused by autoimmune diseases such as Sjögren’s Syndrome, or as a side effect of radiation therapy for head and neck cancer. Generating functional, artificial salivary tissue in vitro, for eventual patient implantation, may be achieved in the future with sophisticated scaffold surfaces that recreate the chemical and mechanical cues of the basement membrane microenvironment [4,5,6]. Biodegradable polymer nanofibers have been used for several decades in the field of tissue engineering as effective high surface-area substrates for encouraging cell attachment, proliferation and differentiation [2]. Nanofiber scaffolds have been developed to act as a substrate primarily for mesenchymal cells [7] but have also been used as a substrate for growth of salivary gland and other types of epithelial cells as a mimic of the basement membrane underlying the surface of the epithelium [4,5,6,8]. Nanofiber meshes resemble the scale and geometry of the Collagen-IV layer, which is a structural component of the basement membrane in natural epithelial systems [8]. In our prior work, the scaffold consists primarily of poly(lactic-*co*-glycolic acid) (PLGA) nanofibers, which are long, thin fibers that form a porous, high surface-area mat. The process of electrospinning is used to generate nanofibers, which entails passing a solution containing a dissolved polymer through a narrow needle aperture into a strong electric field. The field accelerates the fluid towards the collection surface and rapidly precipitates the polymer into an extremely thin and continuous fiber, which then collects the fiber in a random orientation. Our previous studies have explored modifications to the nanofibers such as morphological/biomimetic alterations using craters [4], biomolecular conjugation [6], and modifications to the nanofiber composition [9]. Salivary and epithelial niche specific functional and structural proteins were assayed in order to study the impact of these scaffolds on salivary gland tissue. Western blot analysis was performed for finding bulk concentrations of Aquaporin-5 (AQP5), and immunocytochemistry was used to categorize the lateral membrane proteins as apically-localized, or non-apically localized [4,5,6]. These end-point assays have been useful for identifying superior scaffold characteristics; however, non-destructive methods that can assay live cell cultures are desirable.

## 2. Materials and Methods

### 2.1. Nanoscaffold Transwell Assembly

PLGA nanofibers were prepared in accordance with prior studies [4], using 8 wt % PLGA in hexafluoroisopropanol solution, electrospun at 10 kV at a distance of 15 cm from the collector plate. The syringe pump used was from New Era Pump Systems (Wantagh, NY, USA); 3 mL syringes for pumping the spinning fluid were acquired from Becton, Dickinson and Company (Franklin Lakes, NJ, USA). Screw-on needles of 0.25 mm internal diameter were purchased from EFD (East Providence, RI, USA). Polytetrafluoroethylene tubing for connecting the syringe and spin-head was purchased from VWR (Radnor, PA, USA). The syringe pump was operated at 3 ± 0.2 μL/min volumetric flow rate for 300 min to produce 50 μm thick nanofiber mats. The fibers are deposited onto polycarbonate (PC) membranes (Whatman #110604) at a rate of 3.0 ± 0.5 μL/min, to a thickness of 50 μm after 300 min of electrospinning process time, producing an average fiber diameter of 200 nm. Transwells with a filter diameter 6.5 mm, and growth area of 0.33 cm^2^, designed for 24 well plate equivalence, were purchased from Corning (#3413). In preparation for replacement of the existing membrane, a scalpel is used to slice away the circular membrane, leaving an open aperture at the base of the Transwell. The fibers are cut out using a scalpel and wrapped tightly by hand around the bottom opening of the Transwell. The sheet is then secured around the outside edge of the Transwell using an elastic band. The nanofibers are attached to the top (cell-facing) side of the membrane. The PC membrane is necessary in order to maintain structural stability of the nanofibers above, as they are electrospun directly on to the membrane. An unsupported fiber layer, even of greater thickness, would suffer shape distortions or tearing once immersed and be subject to hydrolysis of the polymer bonds and resultant shear stress. The additional stresses of cell seeding and tissue formation further necessitate the PC underlayer.

Nanofiber scaffolds of 500 μm thickness, produced using a high-throughput bulk synthesis method, and cut into 6.5 mm circular discs were tested in Transwells, on top of existing PC membranes as a proof of concept, and were acquired from Xanofi (Raleigh, NC, USA). However, when tested in the TEER system using submandibular immortalized mouse salivary (SIMS) cells with standard PC Transwells as control, the resulting signal change following cell monolayer coverage was not observed, because the thickness of the layer was too large for ions to fully penetrate at the 40 Hz ECIS setting. Also, thin 10 μm PLGA scaffolds were electrospun for 60 min on to Whatman membranes and used in the Transwells, but over half of the original batch of samples disintegrated following aqueous submersion, via delamination of the nanofibers from the membrane, and subsequent hydrolysis. Finally, 50 μm and 100 μm thick PLGA scaffolds were prepared, and both sample batches managed to remain completely intact during immersion and cell seeding, and also generated the TEER signal response, and would be effective for these trials. For the remainder of these studies, 50 μm scaffolds were chosen due to a shorter preparation time.

### 2.2. SIMS Cell Studies

SIMS cells [10] were used to study the salivary gland tissue in contact with the scaffold substrate. Preparing the substrate for cell attachment required sterilization of dry Transwell under UV light for one hour, followed by incubation in Penicillin–Streptomycin solution (1 mg/mL) for 24 h. Then, scaffold/Transwells were immersed in SIMS media, Dulbecco’s Modified Eagle Medium (DMEM) with pen-step and 10% fetal bovine serum (FBS), for up to 48 h or until substrates appeared fully wetted and transparent to light under magnification. The SIMS cells were seeded on to the substrates at 100,000 cells/well in the upper chamber in a volume of 200 μL, while the lower chamber was filled with 900 μL of cell media. The plate was then transferred into the incubator and allowed to proliferate, while changing media every 1–2 days depending on media coloration. Calcium-free media was composed of calcium-free DMEM and dialyzed FBS.

### 2.3. ECIS-TEER Measurement

The control unit for the ECIS measurements was an ECIS Z-Theta unit from Applied Biophysics (Troy, NY, USA), which connected via a cable linkage to the ECIS 8-well adapter tray. The 8-well Transwell plate for the ECIS system consisted of eight circular fluid wells, with gold electrodes at the base. Grounding electrodes hung down into each well, connected via a continuous metallic top section [3]. The plate was inserted into the adapter tray and several pins were used to secure the well plate and make individual electrical connections to the wells. Once the plate was secured, a time series experiment was begun, which took continuous alternating current impedance measurements throughout the course of the cell experiment. Impedance measurements were gathered at a variety of frequencies, from 40 Hz to 50,000 Hz. One or more wells were left without cells in order to provide a baseline for comparison with the rest of the experimental wells [11].

### 2.4. Immunocytochemistry Staining and Quantification

Immunocytochemistry of SIMS cell layers grown on Transwell membrane inserts was performed in parallel with TEER assays by seeding and incubating cells simultaneously and fixing the cells at the indicated time points. Cells were fixed in 4% paraformaldehyde (PFA) solution in phosphate-buffered saline (PBS) at 4C for 20 min, permeabilized with 0.1% Triton in PBS and stained with anti-Occludin Antibody (Fisher #33150). Anti-mouse A647 was used to fluorescently label the primary antibodies (Jackson #715606150). Following staining, insert membranes were extracted with a scalpel and mounted on cover glass. Confocal microscopy was performed to gather fluorescent volumes, which were then displayed via maximum projection. Total intensity of each image time point was generated by summing the intensity of all pixels within each image using ImageJ.

## 3. Results and Discussion

### 3.1. Transwell Nanofiber Membrane Integration

**Figure 1:**
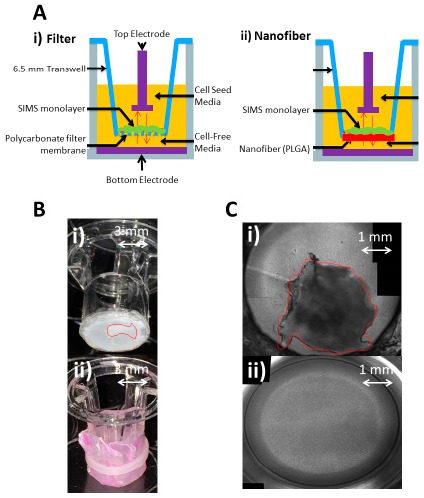
(**A**) Cross-sectional diagrams: (i) Filter-based assembly uses manufactured polycarbonate (PC) filter membrane to support submandibular immortalized mouse salivary (SIMS) epithelial cell monolayer formation, (ii) Nanofiber Transwell, made by excising the filter with a scalpel and securing nanofibers to the base; (**B**) Perspective view of nanofibers added to Transwell inserts by removing the filter membrane with a scalpel: (i) re-attaching with acrylic glue, red outline depicts delaminated nanofibers from the underlying Whatman membrane, (ii) Nanofiber fastening with elastic band; (**C**) 10× optical image top down: (i) using acrylic glue to secure nanofiber, red outline indicates delaminated nanofibers, (ii) using elastic band, fiber integrity is maintained. Band tension provides the ideal combination of force and slippage to support fiber hydration and cell culture.

Previous work has used nanofiber mats to support epithelial cell line growth and polarization, supported by both glass and solid polymer substrates [4,6]. However, these underlying materials are impermeable and are not compatible with a Transwell ECIS application. Eliminating the glass substrate leaves a very thin sheet of nanofibers, which is permeable to diffusion of all media components, providing for the supply of necessary media nutrients to the Transwell cell monolayer as well as facilitating voltage-imposed trans-membrane diffusion. However, lacking a supportive substrate, the fiber mat was observed to deform once immersed in fluid. This is due to residual stress in the fibers retained after the electrospinning process which is released when the aqueous solution hydrolyzes the polymeric bonds. To avoid deformation of the fiber mat, the nanofibers were electrospun on to a layer of filter membrane (Whatman #110605), which is about 10 μm thick and provides physical support for the nanofibers, while still allowing free flow of media. Also, the thickness of previous nanofiber mats on glass or polydimethylsiloxane (PDMS) substrates were about 7–10 µm, which produced a very thin sheet of fibers that ripped easily when handled [8]. For this application, the fiber mat thickness was increased to 50 μm which allows the fibers to survive the removal from the collector material, transfer to the Transwell and attachment to the Transwell. To incorporate the Transwell segment, the filter/fiber stack is then spread over the bottom opening of the Transwell that has had the manufactured membrane already removed. Adhesives were first used to secure the stack to the base of the Transwell, but this resulted in tearing of the nanofiber mat. Small elastic bands to secure the filter/fiber mats are the most effective method, because they provide some tension to effectively secure the fiber mats without damaging them (see Figure 1). This provides enough tension to maintain fiber integrity and base support, while allowing for some movement for contraction in solution.

The integrity of the nanofiber scaffold in the wells was determined with degradation studies in cell media, prior to their use with SIMS cells. Although PLGA nanofibers are biodegradable, and the fibrous structure will deteriorate within two weeks while submerged in cell media, microscope inspection of the scaffolds reveals that the fiber coverage of the well area is maintained. Seeding a salivary gland cell line on these assembled scaffold transwells demonstrated their viability for conducting ECIS– TEER measurements. Thus, an effective strategy to incorporate complex scaffold structures into a biosensor system such as the ECIS–Transwell has been achieved.

### 3.2. Calcium Switch of SIMS Cells and Effect on TEER

**Figure 2:**
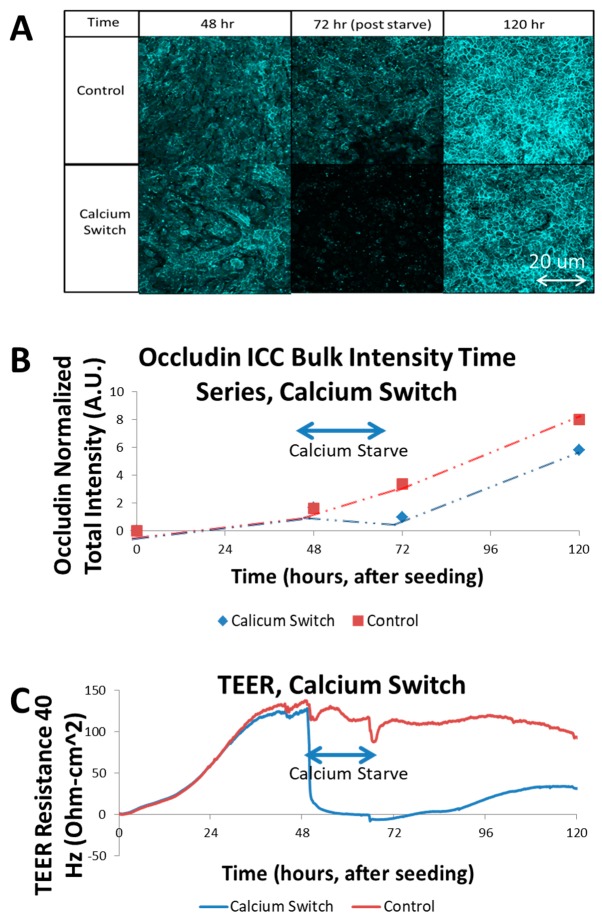
Calcium Switch Experiment trans-epithelial electrical resistance (TEER) vs. intra-class correlation (ICC): (**A**) Chart of immunofluorescence images, generated from maximum projections of Z-stacks of occludin-stained SIMS monolayers on PC membranes extracted from Transwells. Samples were fixed at 48, 72, 120 h. Final (120 h) time point showed greater intensity in control, likely due to continued cellular growth not subject to calcium starvation; (**B**) Graph of total intensity sum (using ImageJ RawInt) of each image, charted with respect to time. Calcium switch occurs at 48–72 h. Intensity rises continuously in control condition, while it is reduced, but then recovers in calcium switch condition; (**C**) Graph of TEER resistance, measured at 40 Hz, in units of Ohm-cm^2^ to normalize for membrane area, although all samples in this study used 0.33 cm^2^ of membrane area per well. Calcium switch drastically reduced TEER signal, which recovered partially after renewing calcium to media.

Calcium is a necessary component for the formation of junctions in epithelial cells. Tight junction proteins, such as occludin, which form the intercellular linkages that act to prohibit free fluid flow along the paracellular pathway, are calcium ion-dependent [12]. In order to demonstrate that the TEER measurement is in fact probing the presence and activity of these tight junction proteins, a calcium-starvation procedure was performed on selected SIMS wells during an ECIS experiment. The cells were allowed to reach confluence on the Transwell membrane in complete media, and then, the media was swapped to a calcium-free media in the selected unmodified PC membrane wells. Post seeding, the resistance increased steadily as the cells attached, proliferated and commenced to cover the membrane, restricting the ionic diffusion between the electrodes to the paracellular pathways of the confluent cells. Once calcium was removed, however, the TEER resistance that had built up rapidly collapsed as the tight junctions disassembled and posed much less of a barrier to ionic species in the media [11,12]. This drop, from a moderate 10 mM calcium concentration, rapidly deprived the calcium-dependent proteins, which lose effectiveness. The result was a rapid drop in TEER resistance (see Figure 2C). The abrupt disturbances in the control TEER signal in Figure 2C were a result of temporary disruption following a cell media change and temperature interruption. The ECIS transfilter assembly must be briefly removed from the incubator at these junctures in order to replace the cell growth media, which causes a noticeable, but short-lived change in temperature. This affects solution conductivity and therefore, impedance. This experimental artifact is mitigated with a cell-free control well to remove thermal and environmental irregularities systematically. Immunocytochemistry for tight junction proteins was used to identify the presence of tight junctions at the apical/paracellular membrane at time points corresponding to the calcium switch assay (Figure 2A). The percent surface coverage of the cell monolayer for the experiments in Figure 2 was verified by confocal microscopy, which was used to detect stained cell nuclei and the actin cytoskeleton and immunostained occludin, a tight junction protein. In all of the well samples the entire scaffold surface was covered in a continuous monolayer. These three-dimensional confocal volumes were superimposed and compressed along the *XY* axis due to the uneven nature of the nanofiber surface (coplanar with the Transwell surface). By comparing the sum of total intensity in each image (Figure 2B) to the TEER resistance, the coinciding effect of calcium deprivation on occludin membrane localization and trans-epithelial diffusion resistance was observed. The control series indicates a continuous increase in occludin intensity over the course of growth time. The samples which undergo the calcium deprivation period show a noticeable reduction in occludin intensity, followed by a recovery of occludin intensity with restoration of calcium. The confocal fluorescence intensity data was also quantified using a simple sum of all field intensities for each volume, resulting in a bulk fluorescence calculation for each sample. These data are presented in Figure 2B, and reinforce the visual finding, which is that the calcium-deprived samples experienced a significant reduction in occludin membrane localization [12]. Thus, the TEER measurements in fact represent the assembly state of the tight junctions by the cells. The purpose of the calcium switch experiment in this study was to identify the maximum range of the TEER signal, to establish an operating window for TEER analysis. This was necessary to validate the ECIS-Z Theta system’s capacity to respond to substantial changes to the cell–cell barrier function. Also, by allowing the TEER signal to increase as the cell monolayer forms and strengthens, and then eliminating the diffusive barrier of tight junctions, if the signal returns to the initial seed baseline level this confirms that the measurement itself is related exclusively to the cell–cell interactions, and not the cell—substrate interactions. If the signal fails to return to the baseline level, then this indicates that some change has occurred in the scaffold itself since seeding, such as severe degradation. This sets up an internal control procedure that could be used in future studies. The calcium switch experiment was run in parallel with ECIS and immunofluorescence to correlate the effect of the calcium deprivation step on both functional tight junction effects in TEER, and structural tight junction effects in occludin levels from fluorescence.

### 3.3. TEER Measurements on Engineered Nanofiber Scaffold

In order to test the functionality of the scaffold Transwells, TEER measurements were taken during a time course cell growth experiment with SIMS cells. The test wells contained PLGA nanofiber mats, with PC 10 μm thick, 0.4 μm pore size cell filter membranes underneath, while the control wells contained only a PC filter membrane. The active wells were seeded with 100,000 SIMS cells per well and immediately placed into the ECIS system within the incubator at 37 °C to begin the experiment. Control wells were not seeded with cells, but filled with fresh SIMS media to account for signal drift and external factors. The impedance at several frequencies between 40 Hz and 50,000 Hz was collected continuously and individually for each well. The frequency used to calculate the TEER was the lowest available one, usually 40 Hz [11]. The barrier function was most pronounced in the resistance data at lower frequencies because the time for charged ions in solution to diffuse across the cell membrane was longer, giving them adequate time to locate the path of least resistance, which was the paracellular pathway between cells and not through the cells themselves. At higher frequencies, the ions were not given as much time and travelled directly through cell membranes more often reducing the selectivity of the signal [1,11].

**Figure 3:**
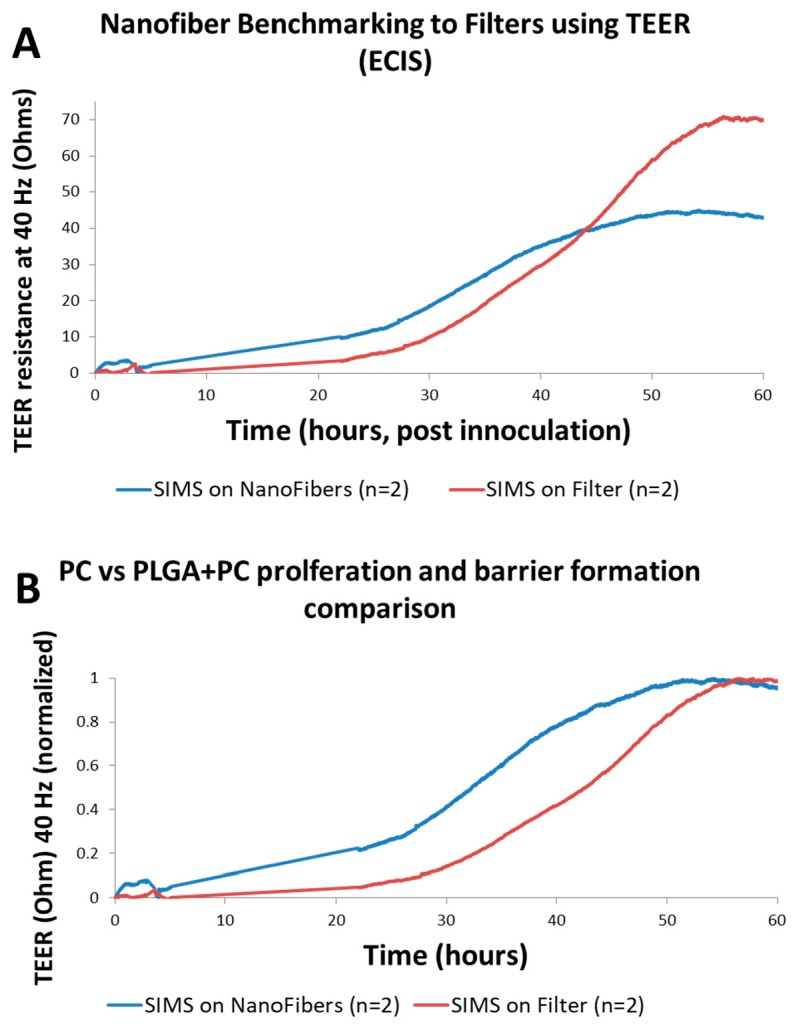
(**A**) Graphs indicating the TEER resistance over time of Transwells with PC membranes vs. PLGA substrates. Similar cellular behavior was observed on both substrates, confirming PLGA substrates are an effective platform for SIMS TEER studies. (**B**) Comparison between unmodified Transwell filter membranes composed of PC 0.4 μm pore size membrane and PLGA nanofibers, using data normalized to the peak signal of each sample.

Comparing the TEER signal of the PLGA fiber Transwells to the unmodified Transwells as seen in Figure 3, both achieved peak resistance in a similar length of time, indicating that monolayer and tight junction development were developing in a similar way. Figure 3B utilizes a normalized dataset, in which the total signal range of each TEER growth curve is normalized to its own maximum. This enables analysis of the relative barrier function growth rate in isolation, despite a difference in saturation value. Figure 3B shows that PLGA scaffolds exhibit a more rapid onset of TEER growth relative to PC scaffolds. The result of this test shows that the characteristic shape of a TEER response through the PLGA scaffold material can be measured, in effect merging the biosensing system into a bioengineered scaffold system. This combination of techniques enables new insights into the complex nanofiber scaffolds.

## 4. Conclusions

In this study, components of the previously proven technologies of ECIS–TEER biosensing and nanofiber cell scaffolds are combined in order to provide a non-destructive assay system for epithelial monolayer formation on nanofiber scaffolds. A Transwell set-up was adapted to allow for inclusion of nanofiber cell scaffolds in the ECIS–TEER system. Immunocytochemistry in the Transwell set-up indicated that the ECIS–TEER measurements correlated with tight junction assembly, and a calcium switch assay used to disrupt tight junctions provided a functional correlation of the ECIS–TEER set-up. This non-destructive assay system can be used for real-time measurements in future studies of scaffold designs to promote epithelial monolayer formation and function.
